# Asexual Reproduction in Holothurians

**DOI:** 10.1155/2014/527234

**Published:** 2014-10-21

**Authors:** Igor Yu. Dolmatov

**Affiliations:** ^1^A. V. Zhirmunsky Institute of Marine Biology, FEB RAS, Palchevsky 17, Vladivostok 690041, Russia; ^2^School of Natural Sciences, Far Eastern Federal University, Suhanova 8, Vladivostok 690950, Russia

## Abstract

Aspects of asexual reproduction in holothurians are discussed. Holothurians are significant as fishery and aquaculture items and have high commercial value. The last review on holothurian asexual reproduction was published 18 years ago and included only 8 species. An analysis of the available literature shows that asexual reproduction has now been confirmed in 16 holothurian species. Five additional species are also most likely capable of fission. The recent discovery of new fissiparous holothurian species indicates that this reproduction mode is more widespread in Holothuroidea than previously believed. New data about the history of the discovery of asexual reproduction in holothurians, features of fission, and regeneration of anterior and posterior fragments are described here. Asexual reproduction is obviously controlled by the integrated systems of the organism, primarily the nervous system. Special molecular mechanisms appear to determine the location where fission occurs along the anterior-posterior axis of the body. Alteration of the connective tissue strength of the body wall may play an important role during fission of holothurians. The basic mechanism of fission is the interaction of matrix metalloproteinases, their inhibitors, and enzymes forming cross-link complexes between fibrils of collagen. The population dynamics of fissiparous holothurians are discussed.

## 1. Introduction

Asexual reproduction is the most ancient mode of reproduction and is observed in representatives of all phyla of modern Metazoa [1–3]. Because asexual reproduction is closely related to the structure of an animal, its types are as diverse as the animals themselves [4]. The variety of manifestations of this phenomenon is even greater because asexual reproduction in different species has different biological functions, such as population growth, regulation of body size, colonization of new sites, and survival under adverse conditions. The evolution of multicellular organisms has apparently passed through repeated losses and restorations of various forms of asexual reproduction [3]. Among modern groups of asexually reproducing invertebrates, holothurians deserve special consideration because of their commercial value.

Holothurians, or sea cucumbers, are a class in the phylum Echinodermata. Holothurians have elongated often worm-shaped bodies that are covered with various outgrowths. Similar to all other echinoderms, holothurians are exclusively marine animals and inhabit all oceans at a broad range of depths, from shallow intertidal zones to 5,000 m and more. Most holothurians are benthic organisms [5, 6], although there are swimming species and most likely completely pelagic ones [7].

Holothurians are significant for commercial fishery and aquaculture. Approximately 66 holothurian species are commonly exploited throughout the world [8–11]. People in these regions consider holothurians not only a traditional commodity but also a commercial resource [9, 12]. Global wild captures and aquaculture production of holothurians during the last 30 years have been increased 7 times amounting more than 20000 t dry weight/annum [12]. Moreover, holothurians are a major source of biologically active substances in biotechnology and medicine [8, 13–16]. They have a wide array of vitamins, minerals, saponins, chondroitin sulfates, polysaccharides, sterols, phenolics, lectins, peptides, glycoprotein, glycosphingolipids, and essential fatty acids [13]. Thus, various aspects of biochemistry, physiology, and developmental biology of these animals are being actively studied.

Holothurians are also notable because they possess diverse regeneration abilities [17–19]. Some species can expel their internal organs, mainly the digestive system, in response to various stimuli and then can quickly restore them [20–24]. Furthermore, many holothurians can regenerate after a transverse cut [25–28].

Some holothurian species are capable of asexual reproduction. Most fissiparous holothurians live in tropical and subtropical zones. The only exceptions are* Ocnus planci* and* O. lactea* which were observed to undergo fission off the coast of la Manche, France [22]. In the southern hemisphere,* Staurothyone inconspicua* also occurs beyond the subtropical zone. This species, with probably dividing juveniles, was collected in Opossum Bay in southern Tasmania [29]. Because of the high commercial value of holothurians, researchers attempt to use their regenerative property and fission ability to develop cultivation methods and increase natural populations [30–36].

The last review on holothurian asexual reproduction was published 18 years ago [37]. This review included only 8 fissiparous species. Since then, asexual reproduction has been observed in additional species (see, e.g., [25, 26, 38]). The discovery of new fissiparous species indicates that this type of reproduction in the class Holothuroidea is more widespread than previously believed. Moreover, new data on the regeneration, population dynamics, and other biological aspects of fissiparous species have been obtained. This information requires systematization. The goal of this review is to analyze the available data on asexual reproduction in holothurians. All the species names used in this paper are provided in accordance with WoRMS (the World Register of Marine Species).

## 2. History of the Discovery of Asexual Reproduction in Holothurians

All currently known fissiparous species of holothurians belong to two orders: Aspidochirotida and Dendrochirotida. Asexual reproduction was first described in dendrochirotids. Dalyell (1851, cited by Monticelli [39]) observed dividing* Ocnus* (as* Cucumaria*)* lactea* and* Ocnus* (as* Cucumaria*)* planci*. Moreover, according to Monticelli [39], Semper described a case of fission in* Havelockia* (as* Cucumaria*)* versicolor*. However, both Dalyell and Semper stated only that fission occurred and did not describe the process. Chadwik (1891, cited by Crozier [40]) provided the first brief description of fission in* O. planci*. Subsequently, Monticelli [39] published a comprehensive study of asexual reproduction in this species.

Benham [41] apparently was the first to provide evidence of asexual reproduction among holothurians from the order Aspidochirotida. He observed four specimens of* Holothuria difficilis* (as* Actinopyga parvula*) which had lighter colored posterior regions and less developed tube feet in a collection of fixed animals from the Kermadec Islands. These animals had regenerated their posterior regions as Benham [41] had believed.

Crozier [40] was the first to directly observe fission in aspidochirotids. He described the fission process in* Holothuria surinamensis* and* Holothuria parvula* (as* Captiva*). Deichmann [42] subsequently confirmed asexual reproduction in* H. difficilis* (as* Actinopyga parvula*).

Another species of fissiparous holothurians was noted only approximately 40 years later. Bonham and Held [43] provided data and a photograph of dividing* Holothuria atra* off the Marshall Islands. Harriott [44] then observed individuals of* Stichopus horrens*,* Stichopus chloronotus*, and* Holothuria edulis* in the field that had undergone fission and were in the process of fission. The specimens of* S. horrens* reported by Harriott [44] most likely belonged to the species* Stichopus monotuberculatus* [45].

In an article describing the neotype for* Ocnus brunneus*, McKenzie [46] indicated that this dendrochirotid species was capable of fission. However, the status of this species was not subsequently confirmed, and the studied individuals most likely belonged to* O. planci* [47]. Subsequently, O'Loughlin and O'Hara [48] discovered the first fissiparous dendrochirotid species during the 20th century. They reported that a new holothurian species,* Squamocnus aureoruber*, from the coastal waters off Australia was capable of asexual reproduction. Conand et al. [49] gave the first description of asexual reproduction for the aspidochirotid* Holothuria leucospilota*, which is widely distributed in the tropical Indo-Pacific Ocean.

Additional fissiparous holothurians were described in the 21st century. The initial four species belong to the order Aspidochirotida. Sonnenholzner [50] reported asexual reproduction in* Holothuria theeli*, which occurs along the coast of Ecuador. Data on the ability of larvae of the sea cucumber* Parastichopus californicus* to bud were also published that year by Eaves and Palmer [51]. This is the only species of holothurian for which asexual reproduction by larvae has been definitely established. There may be other similar species [52]. Japanese researchers then observed an individual of* Stichopus horrens* in the process of fission off Okinawa Island [53]. However, as Byrne et al. [45] believe,* S. horrens* is not capable of fission and the holothurian observed by Kohtsuka et al. [53] was* Stichopus naso*. The ability to reproduce asexually was then established in* Holothuria hilla* [38, 54]. Furthermore, a specimen of* Holothuria portovallartensis* with a growing anterior end was described by Uthicke et al. [55], which most likely indicates the ability to reproduce asexually in this species.

Another three species of fissiparous holothurians belong to the order Dendrochirotida. These species are the Australian* Cucuvitrum rowei* [29] as well as* Cladolabes schmeltzii* and* Colochirus robustus* [25, 26], which occur along the Vietnam coast. Additionally, juvenile* Staurothyone inconspicua* may possess the ability to perform transverse fission [29].

Thus, asexual reproduction has been currently confirmed in 16 holothurian species (see Table 1). Five more species,* Havelockia versicolor*,* Staurothyone inconspicua*,* Stichopus monotuberculatus*,* S. naso*, and* Holothuria portovallartensis*, may also be capable of fission, but further studies are necessary to establish this. Moreover, some holothurian species most likely reproduce asexually in the larval stage [52].

## 3. Asexual Reproduction in the Larval Stage

Currently, there are data for only one holothurian species,* P. californicus*, which can reproduce asexually in the larval stage [51]. In this species, 12.2% of the artificially cultivated larvae underwent fission. The process of asexual reproduction begins in the doliolaria stage. The larvae constrict around the penultimate ciliary band. Subsequently, a deepening of the constriction at the posterior end causes a bud to form. This bud (a daughter organism) retains the ciliary band and remains attached to the mother even after settlement. The bud is separated in the pentactula stage, and a normal Auricularia develops.

In the Florida current of the Gulf Stream System, Balser [52] observed auriculariae of an unknown holothurian species that possessed lateral lobes. Under artificial cultivation the lateral lobes of these auriculariae separated from the individual and formed blastula-like larvae. There was one “hyaline sphere” in the blastocoele. The larvae began gastrulation, but further development then stopped, and the animals died [52].

## 4. Asexual Reproduction in Adults

**Figure 1:**
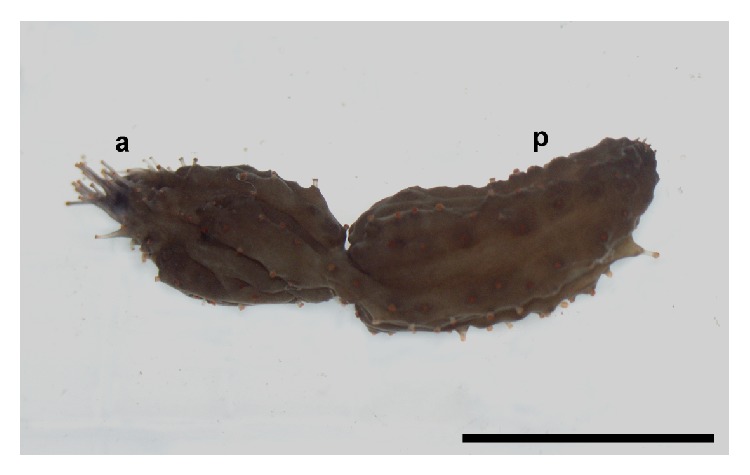
Twisting of* Cladolabes schmeltzii* during fission. a: anterior part; p: posterior part. Scale bar 2 cm.

Asexual reproduction in adult holothurians occurs as transverse fission (architomy) and fragmentation. The most detailed description of the fission process is that by Monticelli [39] who differentiated three fission mechanisms in* O. planci*: by constriction, twisting, or stretching. An analysis of the available fission data on holothurians shows that various holothurian species use different combinations of the methods described by Monticelli (see Table 1). Most holothurians, in which the fission process was observed, began by forming a constriction. Division into fragments proceeds either by the development and deepening of the constriction [49] or as a result of stretching and twisting at the fission site. Usually the posterior sections of the body are attached to a substrate by the tube feet whereas the anterior regions move forward or twist (Figure 1) [26, 56]. The closure of the wound resulting from fission has not been described but probably results from contraction of circular muscles in the body wall.

The duration of the process varies from a few minutes, as in* S. chloronotus* [56], to 1–5 days, as in* H. surinamensis* and* Cladolabes schmeltzii* [26, 40]. The duration of fission most likely depends on the intensity of the transformation of the extracellular matrix of the body wall. For example, the body wall in* S. chloronotus* can quickly soften (J. Lawrence, pers. comm.). Unlike other holothurians, the stimulation of autotomy with potassium chloride in this species causes a rapid softening and rupture of the body wall over a large area and the organs are expelled through the large opening that is formed [26].

The location of the constriction is apparently a species-specific characteristic (see Table 1) [57]. In most of the studied holothurian species, fission occurs approximately across the middle of the body [26, 29, 40, 48, 58–60]. In* H. atra*, the body is divided into two fragments with an anterior : posterior length ratio of 4 : 5 [61]. According to Conand et al. [49], the location of constriction in* H. leucospilota* is shifted toward the anterior end, and the length of the anterior fragments constitutes approximately 19% of the total body length. In* Colochirus robustus*, the ratio of the divided body sections is 2 : 1; that is, the anterior fragment is twice as long as the posterior fragment [25].

Some holothurian species can fragment into several sections of the body simultaneously. This has long been known for species as* O. lactea* and* O. planci*, as described by Dalyell (1851, cited by Monticelli [39]) and Monticelli [39].* Cucuvitrum rowei* also undergoes fragmentation [29]. The repeated division into fragments, which do not completely regenerate the lost section, a process similar to fragmentation, occurs in the aspidochirotid* H. parvula* [60] and the dendrochirotid* Cladolabes schmeltzii* [25].

## 5. Fission Mechanisms

Asexual reproduction in holothurians is a very complex process that involves various mechanisms and organ systems. First, fission is accompanied by complex behaviors, such as stretching and twisting at the fission site. Moreover, according to Purwati [57], holothurians stop feeding prior to fission. Such complex behaviors are obviously controlled by the integrated systems of the organism, primarily the nervous system. This is in accordance with the fact that autotomy, the process which is close to fission, is neurally mediated [65, 66]. Second, fission occurs in certain areas of the body, which is a species-specific characteristic. It means that special molecular mechanisms determine the fission location along the anterior-posterior axis of the body. Third, the body wall, or dermis, in holothurians consists almost exclusively of connective tissue [19, 67]. Therefore, dividing the body by fission is impossible without transforming the extracellular matrix.

Currently, no studies of the contribution of the nervous system to fission regulation and studies of the cellular and molecular mechanisms of fission in holothurians have been done. There are only data on the properties of the extracellular matrix. The connective tissue in holothurians possesses a unique ability to alter its mechanical properties under the effects of various factors [68, 69]. Thus it is called mutable collagenous tissue (MCT) [70] or catch connective tissue [71]. This property apparently is very important for fission [72–75].

The connective tissue of the dermis in holothurians can occur in three states: stiff, standard, and soft [74]. The mechanical stretching of a piece of dermis results in the decreased stiffness of the connective tissue and promotes its transition into the soft state. A mechanism of strain-induced softening at autotomy in holothurians has been proposed [74]. Initially, a small portion of the dermis softens in the area of future rupture. Then, the muscles contract, and the pressure in the coelomic cavity rises. The increased pressure causes an extensive deformation in the softened portion to develop. Stiffness abruptly declines as the threshold value of stretching is surpassed. Simultaneously, an increase in the dissipation (the release of energy by the system) value is registered, which most likely indicates the breakdown of intermolecular bonds. This dissipation enables the dermis to continue deforming at identical pressures (or at lower values). Thus, a positive feedback occurs: the more dermis becomes deformed, the easier it is deformed.

At the cellular level, a special type of cells, juxtaligamental cells, in the connective tissue determines these MCT properties [68]. The cytoplasm of juxtaligamental cells contains numerous granules surrounded by a membrane. These cells are believed to secrete granule-derived substances in response to signals from the nervous system. These substances influence the interaction between molecules in the extracellular matrix of the connective tissue, thus causing rapid changes in the mechanical properties of the latter [76–81].

Immunocytochemistry methods have revealed two proteins in granules of juxtaligamental cells, tensilin and stiparin, which are apparently involved in the alteration mechanisms of MCT properties (Keene, Trotter, unpublished data, cited by Wilkie [69]). These proteins bind to collagen fibrils and are thought to form cross-links between them* in vivo*, thus stabilizing collagen fibers (i.e., fibril bundles). The nucleotide sequence of the transcript of the* tensilin* gene and the predicted amino acid sequence of tensilin are currently known for one holothurian species,* Cucumaria frondosa* [82]. This protein shows a high homology to tissue inhibitors of metalloproteinases (TIMPs), which suggests the participation of matrix metalloproteinases (MMPs) in the functioning of MCT [69]. Studies of compass depressor ligaments in the sea urchin* Paracentrotus lividus* revealed some proteases, which have gelatinase activity [83]. Blocking them with a specific inhibitor increases ligament stiffness. According to the proposed model, the stiffness of MCT depends on interactions between three protein groups, MMPs, TIMPs, and cross-link complexes connecting collagen fibrils to one another [83]. With an increase in TIMP release, MMP is blocked, cross-links develop between collagen fibrils, and MCT is strengthened. The growing concentration or activity of MMP in the extracellular matrix causes the destruction of cross-link complexes. This destruction enables collagen fibrils to slide along one another, which bring MCT into a compliant state. As well as being included in the cross-link complexes, tensilin may be an endogenous MMP inhibitor.

Some internal organs of holothurians such as gut and longitudinal muscle bands (along with body wall) are ruptured during fission. Autotomy of these organs in* Eupentacta quinquesemita* is a result of complete loss in the tensility of their connective tissue [84]. This process is facilitated by muscle contraction. Change in the organs appears to begin from the disruption of the coelomic epithelium. Then the connective tissue is infiltrated by coelomic fluid. It is proposed that the coelomic fluid of holothurians contains evisceration factor which affects connective tissue provoking loss of its tensility [66]. In contrast to other echinoderms autotomy (evisceration) in holothurians may occur without participation of juxtaligamental cells [84]. The processes of these cells remained largely intact despite extensive breakdown of internal organs.

A similar mechanism that alters the connective tissue stiffness of the dermis may also operate during asexual reproduction of holothurians. During fission, a local softening of the dermis because of MMP activity occurs at a certain site along the anterior-posterior axis of holothurians. This process might be accompanied by contractions of the radial muscles in the body wall, producing additional tensile. A prolonged local effect on the dermis would cause even greater softening, and a constriction in the body wall would be formed. Additional twisting or stretching body movements would accelerate fission. The difference in fission duration between various fissiparous species is most likely determined by differences in extracellular matrix properties and MMP, TIMP, and other enzymatic activities.

## 6. Regeneration of Internal Organs after Fission

**Figure 2:**
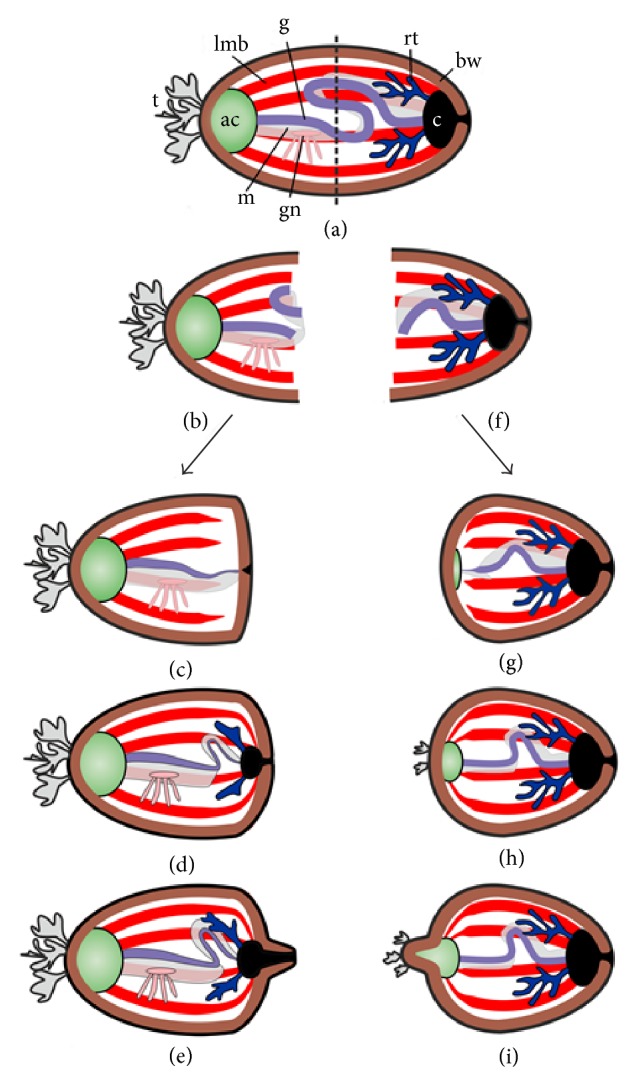
Scheme of regeneration of internal organs after fission in holothurians. (a) Animal before fission. (b) Anterior fragment just after fission. (c) Formation of gut and cloaca in anterior fragment. (d) Formation of respiratory trees in anterior fragment. (e) Growth of the posterior part of the body. (f) Posterior fragment just after fission. (g) Formation of AC and gut rudiments in posterior fragment. (h) Posterior fragment with regenerated internal organs. (i) Growth of the anterior part of the body. ac: aquapharyngeal complex; bw: body wall; c: cloaca; g: gut; gn: gonad; lmb: longitudinal muscle band; m: mesentery; rt: respiratory tree; t: tentacles. Dotted line: site of division of the body during fission.

The set of organs remaining in the fragments after fission is approximately identical for all studied fissiparous holothurian species (Figures 2(a) and 2(b)). The differences are related only to the location of the fission site. In* H. atra*,* H. parvula*,* S. chloronotus*,* Colochirus robustus*, and* Cladolabes schmeltzii*, which perform fission in the middle or posterior portions of their bodies, the anterior fragment retains the aquapharyngeal complex (AC), gonads, and one or two segments of the gut [25, 26, 58, 60, 85]. The posterior fragment of these animals contains the cloaca, a larger portion of the gut, and the respiratory trees. The posterior fragment of aspidochirotids of the family Holothuriidae also contains Cuvierian tubules. In* H. leucospilota*, which divides closer to the anterior end, the gonad may remain in the posterior [49] or anterior fragment [57].

There are two variants of division of the intestine, which are most likely depending on the position of the intestine in the body cavity at the moment of fission [86]. If the intestinal loops are located posteriorly the fission site, rupture occurs only in the middle of the first descending portion of the intestine (1DP). If the intestinal loops cross the fission plane, the digestive tube is divided into three places, and two fragments remain in both sections of the animal.

### 6.1. Macromorphological Features of Regeneration

Regeneration of the internal organs after fission has been described in varying degrees of details for 6 holothurian species:* H. difficilis* [42],* H. parvula* [60],* H. atra* [85, 86],* H. leucospilota* [49],* S. chloronotus* [58], and* C. schmeltzii* [26, 87]. All descriptions are based only on analyses of fragments collected in nature, and, thus, the duration of regeneration remains unknown. All data assume that the regeneration of the internal organs after fission is similar in all holothurians, at least at the macromorphological level (Figure 2).

In the anterior fragment, regeneration after fission begins with partial atrophy of the damaged 1DP, the length of which is consequently reduced. Simultaneously, the wound at the posterior end is repaired. Moreover, the isolated fragment of gut, if present, is broken down. The end of 1DP then begins to grow backward, down the mesentery, thus forming the primordium of the intestine. Simultaneously, the cloaca develops at the posterior end of the anterior fragment. The primordium of the intestine becomes longer and grows into the cloaca, and the integrity of the digestive system is restored (Figure 2(c)). Animals at this regeneration stage restore the terminal regions of the longitudinal muscle bands (LMB) that were damaged during fission. LMB ends become thinner and grow toward the cloaca. After the intestine and cloaca merge, the primordia of the respiratory trees appear on the dorsal side of the cloaca (Figure 2(d)).

Body growth also begins at this stage. At the posterior end, a small outgrowth emerges and subsequently becomes longer, thus forming the posterior region of the animal (Figure 2(e)). Respiratory trees develop with the growth of the posterior end. The respiratory trees gradually grow and form lateral branches. In these animals, the terminal regions of the LMB remain thinner than normal. The restoration of LMB is most likely completed later.

The main event in the posterior fragment is the regeneration of the AC. First, a connective tissue swelling, which represents the AC primordium, is formed at the anterior end of the animal between the torn ends of ambulacra (Figure 2(g)). Then, the terminal regions of the radial nerve cords and radial water-vascular canals grow into AC primordium. Subsequently, these regions form the nerve ring and circular water-vascular canal, respectively, around the AC. The torn anterior region of the intestine is transformed along with the development of the AC. At the damage site, the intestine becomes thinner and begins growing forward up the mesentery (Figure 2(g)). The primordium of the intestine, in the shape of a thin tube, grows into the AC and the integrity of the digestive system is restored (Figure 2(h)). Then the animal begins to grow. At the anterior end, a small outgrowth emerges and gradually grows longer, thus forming the anterior region of the animal (Figure 2(i)).

### 6.2. Microanatomical Features of Regeneration

Regeneration after fission at the cellular level has been described only for* C. schmeltzii* [87]. Here, the distinguishing feature of development of the digestive system in both fragments is the formation of the intestine from two primordia. The entodermal region (intestine) is formed as a result of dedifferentiation and the migration of enterocytes of the remaining 1DP. During dedifferentiation, the enterocytes lose many secretory granules and microvilli, and their height decreases. Nevertheless, the enterocytes retain their intercellular junctions. The luminal epithelium of the intestine is not broken down. After merging with the cloaca (anterior fragment) or AC (posterior fragment) and restoring the integrity of the digestive tract, enterocytes become specialized depending on their position along the intestine [87]. In general, the regeneration mechanisms of the intestine in* C. schmeltzii* during asexual reproduction are similar to those of intestinal regeneration after autotomy in other holothurian species [18, 21, 23, 88–91]. In both cases, the lost regions are formed through dedifferentiation and migration of the remaining cells of the luminal epithelium.

Ectodermal sections (the pharynx, esophagus, and posterior end of cloaca) are apparently formed from epidermal cells which migrate from the epidermis of the body wall of the animal into the connective tissue of the body wall and AC primordium. In this case, the cells most likely retain links with one another, and their intercellular junctions are not broken. In the early stages of gut regeneration, these cells begin synthesizing the cuticle. Subsequently, the ectodermal region merges with the entodermal region; thus, the integrity of the digestive system is restored.

Respiratory trees in the anterior fragment develop through the transformation of the dorsal wall of the anterior region of the cloaca. The distinguishing regeneration feature of the respiratory system is the rapid specialization of cells in the luminal epithelium. These cells have lamellae on the apical surface and are connected with one another through a complex of specific intercellular junctions during the early stages of formation. Therefore, the regeneration of respiratory trees in* C. schmeltzii* is similar to the development and regeneration of these organs after evisceration in* Apostichopus japonicus* [92, 93].

New muscle bundles are formed in the regenerated ends of LMB from the coelomic epithelium, which covers the muscles. First, groups of cells are embedded in the connective tissue. After that these cells transform into myocytes, and myofilaments are observed in their cytoplasm. These groups of cells are then separated from the epithelium and form new muscle bundles. This process is identical to muscle growth and regeneration in echinoderms [94–98].

The regeneration of the AC in the posterior fragment also occurs from cells in the remaining organs [87] and is similar to repair after evisceration [18, 99]. The cells of the terminal segments of the radial water-vascular canals and radial nerve cords are dedifferentiated and migrate down the connective tissue primordium of the AC. The contractile apparatus is broken down in the myoepithelial cells of the luminal epithelium of the water-vascular canals. Myofilaments aggregate into spindle-like structures and are ejected from the cytoplasm. Intercellular junctions are not broken during dedifferentiation, and the cells migrate within the epithelium. Therefore, the tubular primordium of the water-vascular canal forms and gradually grows along the AC. The nerve cords apparently grow in a similar manner. Both neurons and glial cells participate in nervous system regeneration.

In general, regeneration of the internal organs after fission in* C. schmeltzii* is similar to regeneration of these structures after evisceration or artificial damage. Transformation of the remaining sections of organs plays a major role in restoration. The main mechanisms are dedifferentiation and the relocation of epithelial layers (epithelial morphogenesis).

### 6.3. Growth of the Body

Growth of the body begins when the internal organs are formed (Figures 2(e) and 2(i)). Initial signs of regeneration appear at this stage in most holothurians. At the fission site, the dermis is depigmented and a protuberance forms. The growth duration of the body varies broadly and apparently depends on the species. In* C. schmeltzii* individuals, which were most likely caught soon after fission and did not have visual signs of growth at the end, a 2-3 mm long outgrowth (10–15% of body length) formed within 25 days when the animals were maintained under artificial conditions (Dolmatov, unpublished data). In* S. chloronotus*, growth of the external region to normal size required up to one month [59, 64]. According to Jaquemet et al. [100] the regeneration of* H. atra* after fission took about six months.

Assessments of fission and regeneration rates are typically based on the external morphology of the animals, particularly the presence of the growing anterior or posterior end of the body. In this case, all the animals, which did not manifest a distinct outgrowth at the fission site, were combined into one group and considered as just divided individuals. These holothurians were used for the fission intensity assessment [49, 57, 85, 86]. But, in fact, this group comprises holothurians at various regeneration stages [60, 85, 87]. The duration of these stages in nature is unknown, and estimations in holothurians can be only indirect and based on regeneration experiments after artificial cutting. We may assume that the development of organs during asexual reproduction and after artificial cutting progresses at identical rates. In* C. schmeltzii*, the formation of internal organs without the growth of an external region occurs for approximately 30 days [26]. Consequently, in animals that have a small outgrowth at the posterior end, fission occurred over a month ago. The duration of regeneration of internal organs in other holothurians may range from 1.5 to 3 months [26, 33, 35]. Thus, animals with small outgrowths could have divided a few months ago. This fact should be considered when assessing the seasonal intensity of fission.

### 6.4. Gonad Development

Gonad development is an important matter in the study of holothurian asexual reproduction because one of the fragments after fission completely loses its reproductive system, including the set of primary germ cells. Gonad regeneration apparently occurs in all the fissiparous holothurians, as even populations with a high degree of fission continue to reproduce sexually, and the proportion of asexual individuals does not increase [59, 60, 64].

The development of the reproductive system after fission has been studied for only one species,* H. parvula* [27]. The gonads formed late. The primordium of the reproductive system was observed only in those individuals that had completely formed the rest of the organs. The base of the gonad develops first as one or several aggregations of cells in the intestinal mesentery. A histological analysis showed that the base of the gonad contained primary germ cells. The gonadal tubules then begin growing from the gonad base. During development, the gonoduct begins to form and grows from the gonad to the dorsal region of the body wall.

## 7. Artificial Fission

The so-called “artificial fission” deserves special consideration. “Artificial fission” is division of a holothurian into two sections by constricting the body with a rubber band [30, 32, 34–36] or by transverse cutting [31]. Monticelli [39] conducted the first such experiments. The artificial division of a holothurian into two sections is very far from natural stimulation of asexual reproduction and could be considered as only an imitation of fission. When a rubber band was used, behavioral reactions related to fission (constriction, twisting, and stretching) were absent [32]. Most likely, no internal mechanisms triggering fission were involved. Simple cutting of an animal has even less in common with natural fission. Nevertheless, such studies are important and useful to learn more about regeneration abilities to develop cultivation technologies and increase holothurian populations. Transverse cutting experiments have shown that not all holothurians can restore both body fragments [18, 26, 28, 35, 36]. For example, both anterior and posterior fragments of* Actinopyga mauritiana* die after constricting of the body with a rubber band [35]. In* Holothuria fuscogilva*,* A. miliaris*, and* Stichopus variegatus* only the posterior parts can regenerate into whole animals [35]. On the other hand smaller (younger) individuals of* A. mauritiana*,* H. fuscogilva*, and* S. variegatus* have higher survivorship and shorter regeneration time relative to adults [36]. After transverse cutting both fragments of* Holothuria pervicax*,* H. impatiens*, and* Massinium magnum* die [25, 26]. Only posterior parts of* Apostichopus japonicus*,* Holothuria scabra*,* Ohshimella ehrenbergi*, and* Colochirus quadrangularis* can regenerate lost anterior structures after such operation [18, 25, 26].

## 8. Population Dynamics

### 8.1. Seasonal Features and Relationship with Sexual Reproduction

Holothurian asexual reproduction may occur throughout the year with activity varying between populations and seasons. In populations of* H. atra* [44, 64, 101–103],* S. chloronotus* [59],* H. edulis* [64], and* H. difficilis* [63], living in the southern hemisphere, the highest fission activity was observed during winter when the water temperature was the lowest. The peak of sexual reproduction in these species occurred during the warm period of the year (November to February). In the northern hemisphere, the highest fission activity was recorded within the summer months and coincided with the sexual breeding season [60, 62, 104]. These differences in the fission season between the northern and southern hemispheres could be caused by differences in timing of mid-day low tides in the summer and winter [62].

It is obvious that asexual reproduction has a negative effect on sexual activity. After fission, one-half of the animals (mainly the posterior fragment) do not have gonads. Correspondingly, the proportion of sexual individuals declined in populations of even those species that had both types of reproduction that was very synchronized and in different seasons [59, 60]. A typical example of the negative influence of fission on sexual reproduction is the population of* H. atra* along the coasts of Taiwan. Because the sexual breeding season coincides with the season of maximum fission, the two halves produced by fission have no gonads and do not spawn [62, 104]. In* H. parvula*, the proportion of individuals with mature gonads does not exceed 10% during the year [60].

A decrease in reproductive potential is manifested not only as a low gonadal index but also as a biased sex ratio with a prevalence of males and smaller body sizes. For some populations of fissiparous holothurians, a bias in sex ratio is typical [39, 44, 59, 63, 105, 106]. Thus, the male to female ratio may reach 31 : 1 [59]. Asexual reproduction is believed to provoke a biased sex ratio in echinoderms [106–108]. According to McGovern [109], one of the main causes of this phenomenon is a difference in the fission rate between males and females. However, this relationship is not as unambiguous as it may appear. There are also populations of fissiparous species, in which the sex ratio is 1 : 1 [59]. Moreover, the sex ratio may vary with age. According to Harriott [44], males prevailed (8.5 : 1) among* H. atra* individuals that weighed less than 100 g. The proportion of females increased as body weight increased. The male to female ratio among animals of more than 1,000 g was 0.7 : 1. In the* H. parvula* population in Bermuda, a sex ratio bias was apparently absent [60]. In a population of* Cladolabes schmeltzii* from Nha Trang Bay, the number of females was more than males (Kamenev and Dolmatov, unpublished data). There are numerous causes of a biased sex ratio, which may differ between species and populations [109–112]. However, no special studies of biased sex ratio causes in holothurians have been conducted. Uthicke et al. [106] noted that a decrease in female proportion might result from a higher mortality among adult females, higher fission rate among males, lower sexual recruitment, or higher mortality among larval females.

A high intensity of fission causes a gradual decline in average body weight and size [39, 59, 75]. The subsequent fate of a population apparently depends on the growth rate of fragments and environmental conditions [102]. According to Monticelli [39], repeated fission in* O. planci* resulted in a decrease in individual's body size and death. The mean weight of* S. chloronotus* in some populations at La Réunion decreased as much as 1.5 times (from 55 to 37 g) over three years of observation [59]. However, as was shown in Great Barrier Reef populations, body weight can be restored to normal within a few months [75].

### 8.2. Population Genetics

Very little is known about the genetic structure of populations of fissiparous holothurians. The structure has been studied in only two species,* H. atra* [105, 113] and* S. chloronotus* [106, 113, 114]. Asexual reproduction has a considerable impact on the genetic structure of populations of these species [114]. Genetic diversity is reduced in fissiparous holothurians. In the most studied population, 40–60% of individuals resulted from asexual reproduction [105, 106, 113]. In some populations of* S. chloronotus*, which are nearly entirely composed of males, only up to 20% of all individuals were sexually produced [114]. The “genetic link” between populations occurred only by sexually produced larvae. Holothurian clones are restricted to local populations [114].

## 9. Factors That Influence Asexual Reproduction

The intensity of asexual reproduction varies greatly between populations of the same holothurian species [59, 75]. This indicates that the environment plays a major role in triggering and regulating fission [59]. The factors that influence asexual reproduction in holothurian larvae are unknown. Nevertheless, there are experimental data on other echinoderms that serve as the basis for two proposed mechanisms for the stimulation of asexual reproduction. Sea star and sea urchin larvae, when cultivated at the optimum temperature and with diverse food resources, undergo cloning at a higher rate [115, 116]. Most likely, the larvae that were under the most suitable habitat conditions were stimulated to asexually reproduce. Growth in a number of individuals living under optimum conditions apparently increases the probability of successful development, metamorphosis, and reaching the juvenile stage that eventually results in the growth of population size.

However, in the sea urchin* Dendraster excentricus* the rate of asexual reproduction in pluteus larvae increases when the external mucus from fish (predator cues) is introduced into the cultivation medium [117–119]. This behavior is a predation avoidance reaction. The effect of mucus activates budding and fission in larvae. Therefore, body size decreases, which enables individuals to escape from predators more successfully [118].

In adult holothurians, the main factors that influence the asexual reproduction rate are low environmental stability, high mortality, small individual body size, and low sexual reproductive activity [37, 75, 102, 103]. Emson and Wilkie [22] noted that many fissiparous brittle star species inhabit intertidal or shallow waters. In this environment, brittle stars are exposed to greatly varying environmental factors that may trigger asexual reproduction. In* Colochirus robustus*, fission is most likely stimulated by stress when they are maintained under unsuitable conditions [25].

Environmental influences on intensity of fission have also been demonstrated for* H. atra*. This species was observed to have two size morphs. Small individuals can reproduce both sexually and asexually whereas large individuals reproduce only sexually [62, 63, 104]. Both sizes are observed in the same species and represent phenotypic ecotypes [11]. Small fissiparous individuals inhabit the intertidal zone, which is characterized by significant variations in environmental conditions, whereas large individuals are adapted to the more stable subtidal zone [62, 75, 85, 102, 104, 105, 120]. If small individuals are relocated from intertidal to subtidal habitats, they become big [102, 104]. Moreover, higher food availability, because of decreased population size, stimulates the growth of individuals and stops asexual reproduction [102, 103].

The size of some populations of fissiparous holothurians, despite a high fission rate, can remain constant for a long time [44, 75, 85, 101–104, 120, 121]. This scenario indicates that some individuals are eliminated from this population because of mortality after asexual reproduction or emigration to other habitats [44, 85, 102]. According to the model proposed by Uthicke [75], population size stability indicates a feedback mechanism between asexual reproduction and mortality and emigration. This mechanism maintains population density near average values, which fulfill the potential of the environment.

## 10. Conclusion

An analysis of the available literature shows that asexual reproduction has been documented for 16 holothurian species (see Table 1). Five additional species are also most likely capable of fission but more studies are necessary to confirm this. Moreover, some holothurian species may reproduce asexually in the larval stage [52]. The recent discovery of new fissiparous holothurian species indicates that this reproduction mode is more widespread in Holothuroidea than previously believed.

Undoubtedly, asexual reproduction plays a major role in the life activity of holothurians and supports population size. Fission acquires particular significance for commercially valuable species that are exposed to widespread overfishing [12]. Active fission enables holothurians to support a large population and mitigate negative external effects. However, although there are some advantages under certain conditions, cloning most likely is only beneficial for the short term. A decline in body size and sexual reproduction activity for a long time may decrease population size and survival of the species. Unlike other clonal animals, such as Cnidaria, the development of internal organs and further growth in holothurians may be for long term (over several months). The gonad forms only several months after fission. Therefore, intensity of sexual reproduction decreases, and a bias in the sex ratio most likely occurs.

It is evident that the adaptive importance and advantages of asexual reproduction in holothurians can be related to its combination with sexual reproduction. The presence of two ecomorphs is a notable adaptation because it enables animals to use completely different habitat conditions. Moreover, asexual reproduction occurs as a mechanism to support large population sizes, where larval emigration is significant [75, 122].

Many problems regarding asexual reproduction in holothurians have still to be solved. In particular, there are no studies of the cellular and molecular mechanisms of fission. Currently, which factors (genes) determine the location of the site where the body of a holothurian will divide remains unknown. The matters concerning the restoration of the reproductive system remain unstudied. The source of primary germ cells in fragments that lack gonads is also unclear. Moreover, additional studies of factors that stimulate fission and increase asexual reproduction activity in a population are necessary.

Asexual reproduction in holothurians is a notable and poorly investigated phenomenon. The study of fission and its associated regeneration is essential for understanding the reproduction of holothurians. It is possible that the development of methods to stimulate asexual reproduction or regeneration after artificial cutting will be helpful to restore holothurian populations and provide additional economic effects.

## Figures and Tables

**Table 1:** Features of asexual reproduction in holothurians.

	Fission method	Site of fission (anterior/posterior)	Duration of fission	Bias sex ratio	Comments
Aspidochirotida					
Holothuriidae					
*Holothuria atra *	c/t/s [62]	4 : 5 [61]	n	m > f [44]	
*H. difficilis *	n	n	n	m < f [63]	
*H. edulis *	t/s [64]	n	n	n	
*H. hilla* [38]	n	n	n	n	
*H. leucospilota *	c [49]	1 : 4 [49]	n	n	
*H. parvula *	n	1 : 1 [60]	n	n	
*H. portovallartensis* [55]	n	n	n	n	Confirmation is needed
*H. surinamensis *	c [40]	1 : 1 [40]	1–5 d [40]	n	
*H. theeli* [50]	n	n	n	n	
Stichopodidae					
*Stichopus chloronotus *	c/s [56]	1 : 1 [58]	5 min [56]	m > f [59]	
*S. monotuberculatus* [45]	n	n	n	n	Former *S. horrens* [44], confirmation is needed
*S. naso* [45]	n	1 : 1	n	n	Former *S. horrens* [53], confirmation is needed
*Parastichopus californicus *	b [51]				Larvae

Dendrochirotida					
Cucumariidae					
*Cucuvitrum rowei *	n	fr [29]	n	n	
*Ocnus lactea *	n	fr^1^	n	n	
*O. planci *	c, s, t [39]	fr [39]	14 h [39]	m > f [39]	
*Squamocnus aureoruber *	c/s [48]	1 : 1 [48]	n	n	
*Staurothyone inconspicua *	c [29]	1 : 1 [29]	n	n	Confirmation is needed
*Colochirus robustus *	c/s [25]	2 : 1 [25]	~24 h [25]	n	
Sclerodactylidae					
*Cladolabes schmeltzi *	c/s [26]	1 : 1 [26]	24 h [26]	m < f^2^	
Phyllophoridae					
*Havelockia versicolor* ^3^	n	n	n	n	Confirmation is needed

b—budding; c—fission by constriction; f—female; fr—fragmentation; m—male; n—no data; s—fission by stretching; t—fission by twisting.

^1^Dalyell, 1851, cited by Monticelli [39]; ^2^Kamenev and Dolmatov, unpublished data; ^3^Semper (cited by Monticelli [39]).
